# Carbonization of Corn Leaf Waste for Na-Ion Storage Application Using Water-Soluble Carboxymethyl Cellulose Binder

**DOI:** 10.3390/gels9090701

**Published:** 2023-08-30

**Authors:** Ruiping Li, Ali Reza Kamali

**Affiliations:** Energy and Environmental Materials Research Centre (E2MC), School of Metallurgy, Northeastern University, Shenyang 110819, China

**Keywords:** corn, biomass, carbonization, Na-ion battery, anode

## Abstract

Hard carbon materials are considered to be the most practical anode materials for sodium ion batteries because of the rich availability of their resources and potentially low cost. Here, the conversion of corn leaf biomass, a largely available agricultural waste, into carbonaceous materials for Na-ion storage application is reported. Thermal analysis investigation determines the presence of exothermic events occurring during the thermal treatment of the biomass. Accordingly, various temperatures of 400, 500, and 600 °C are selected to perform carbonization treatment trials, leading to the formation of various biocarbons. The materials obtained are characterized by a combination of methods, including X-ray diffraction, electron microscopy, surface evaluation, Raman spectroscopy, and electrochemical characterizations. The Na-ion storage performances of these materials are investigated using water-soluble carboxymethyl cellulose binder, highlighting the influence of the carbonization temperature on the electrochemical performance of biocarbons. Moreover, the influence of post-mechanochemical treatment on the Na-ion storage performance of biocarbons is studied through kinetic evaluations. It is confirmed that reducing the particle sizes and increasing the carbon purity of biocarbons and the formation of gel polymeric networks would improve the Na-ion storage capacity, as well as the pseudocapacitive contribution to the total current. At a high-current density of 500 mA g^−1^, a specific Na-ion storage capacity of 134 mAh g^−1^ is recorded on the biocarbon prepared at 600 °C, followed by ball-milling and washing treatment, exhibiting a reduced charge transfer resistance of 49 Ω and an improved Na-ion diffusion coefficient of 4.8 × 10^−19^ cm^2^ s^−1^. This article proposes a simple and effective technique for the preparation of low-cost biocarbons to be used as the anode of Na-ion batteries.

## 1. Introduction

The low-cost and efficient electrification of various sectors is an essential global measure to achieve targets of sustainability [1,2]. Although mainstream energy storage systems are currently based on lithium ion batteries (LIBs), the development of alternative systems, such as sodium ion batteries (SIBs), are becoming significantly important due to the foreseeable obstacles associated with LIBs [3,4]. Among them, the supply of lithium resources may be exhausted in future due to the widespread electrification of various sectors [5]. Consequently, the development of simple and effective ways of preparing materials for Na-ion storage applications is of significant scientific and commercial importance. In particular, commercial SIBs require low-cost anode materials [6,7,8] with extensive availability of resources, non-toxicity, and specific electrochemical characteristics, such as sufficiently low Na-ion insertion/extraction potentials and high specific capacity. Among various materials evaluated as the anode of SIBs, hard carbons may provide the specifications mentioned above; therefore, they have become the most widely characterized anode materials for NIBs, with the highest possibility of commercialization [9,10]. Disordered pseudo-graphitized and defective domains in such hard carbons provide an efficient platform for Na insertion/extraction.

Among hard carbons, those extracted from environment-friendly and low-cost biomass waste can be highly desirable choices [11]. Therefore, biomass wastes such as coconut shells, walnut shells, and corn silk [12] have been used as the carbon source for the preparation of hard carbons for Na-ion storage. The carbonization of such materials is performed by grinding the biomass, followed by thermal annealing under an inert atmosphere (Ar) to 1300 °C with a dwell time of around 1 h, and washing with HCl solutions for several hours to remove impurities [12]. Zhu et al. [13] carbonized corn pods at 700 °C for 2 h under N_2_ gas, followed by washing with HCl solutions and treating with a mixture of Cu(NO_3_)_3_, Co(NO_3_)_2_, NH_4_F, and urea at 120 °C (8 h) to prepare a CuCo_2_O_4_/corn pod structure, which was subsequently used as the anode of SIBs. To fabricate such electrodes, polyvinylidene fluoride (PVDF) is often used as the binder; therefore, the utilization of organic solvents such as N-methyl-2-pyrrolidone (NMP) becomes inevitable due to the lack of solubility of PVDF in water. Due to the toxic nature of NMP, the utilization of water-soluble binders such as carboxymethyl cellulose (CMC) would be of significant environmental and economic importance.

It should be mentioned that, in aqueous slurries, carbon particles tend to aggregate with each other due to their hydrophobicity, reducing the homogeneity of the slurry. The addition of CMC can induce the dispersion of the carbon particles by adsorbing onto the carbon surfaces, while the remaining CMC can form gel polymeric networks [14]. Park et al. [15] studied the influence of CMC binder on rheological properties of anode slurries made of carbon materials, and found that, at sufficiently high concentrations, CMC acts as both a dispersant and a gelling agent, forming a fibrillar network. Other observations suggest that the gelatinization behavior of CMC and alternative water-soluble binders such as guar gum influences the electrochemical performance of anodes used in metal-ion cells [16].

On the other hand, despite its availability, corn leaf waste has not been used as the precursor material for Na-ion storage applications, while some other applications have been studied using corn leaf waste. For example, Amer et al. [17] used Jordanian corn leaf waste through pyrolysis at 300–450 °C under nitrogen to prepare biofuels. Corn leaf can also be considered as an interesting source to fabricate Na-ion storage materials. However, considering that the application of corn leaf waste for the preparation of anode materials for SIBs has not been investigated in the literature, this study aims at studying the effect of carbonization temperature and mechanochemical activation on Na-ion storage performance of corn leaf. It provides experimental evidence on the feasibility of utilizing such a highly available biomass for Na-ion storage application through mild processing conditions, without unitizing potentially problematic/toxic chemicals, using a water-soluble CMC binder.

## 2. Results

**Figure 1:**
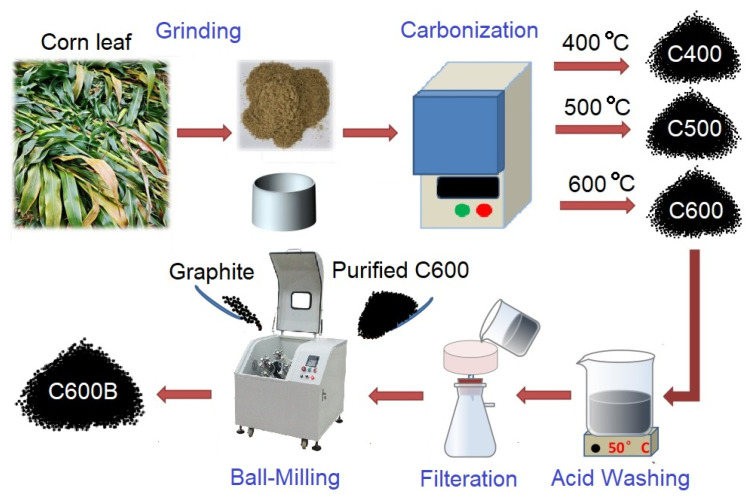
Illustration of the method employed for the preparation of samples using corn leaf biomass resource. The influence of carbonization temperature and the mechanochemical modification on the Na-ion storage performance of products are studied.

Firstly, in this research, the critical temperatures that appeared during the carbonization heat-treatment of powdered corn leaf waste were identified using thermal analysis. Then, carbonization was conducted in a muffle furnace at selected temperatures of 400, 500, and 600 °C, and the Na-ion storage performances of the products were investigated. Moreover, the sample prepared at 600 °C was modified by a mechanochemical process to investigate the effect of such treatment on the electrochemical properties of the product. Figure 1 illustrates the method employed for the preparation of samples.

### 2.1. Thermal Analysis

**Figure 2:**
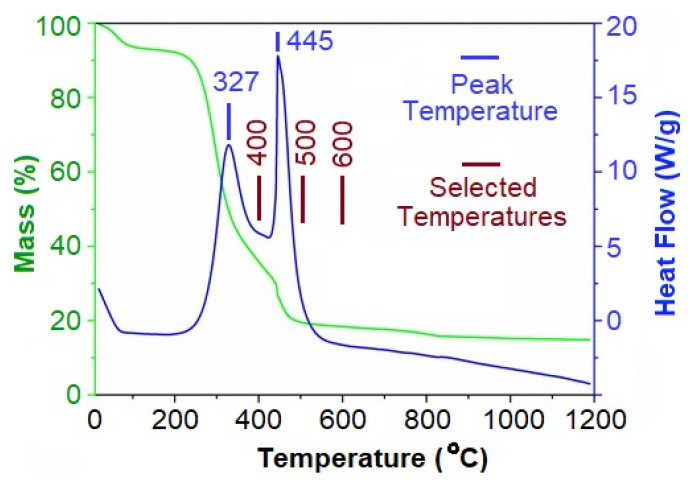
TGA-DSC profiles of the corn leaves at a heating rate of 20 °C min^−1^, recorded at an air flow rate of 100 mL min^−1^. The peak temperatures and temperatures selected to perform the heat-treatment trials are also exhibited. Upward peaks in the DSC curve exhibit exothermic events.

Thermal analysis is an effective tool to study thermally induced reactions [18,19]. On this study, differential scanning calorimetry (DSC) and thermogravimetric analysis (TGA) were conducted on the corn leaf powder at 20 °C min^−1^ under an air flow rate of 100 mL min^−1^ to shed light on the thermal degradation of the biomass, and the results obtained are shown in Figure 2. As can be observed from the TGA curve of Figure 2, the minor mass loss of around 3.5 wt% observed below 100 °C is related to the loss of moisture and other volatiles. In contrast, there is a major mass loss of ≈65.0 wt% at 100–440 °C. The mass loss observed is accompanied by the exothermic peak of 327 °C, according to the DSC curve. This event can be related to the thermal decomposition of the hemicellulose of leaves, which typically occurs at 280–500 °C [20,21]. Another mass loss of 14.5 wt% can be detected, according to the TGA curve of Figure 2, at temperatures greater than 440 °C. This mass loss is accompanied by an exothermic peak at 445 °C, according to the DSC curve. This event can be related to a combination of reasons, including the decomposition of lignin present in the biomass, the removal of functional groups, and the reorganization of the carbon structure [22]. A remaining residue mass of around 17% can be identified, based on the TGA curve of Figure 2, at temperatures greater than 500 °C. The remaining material is relatively thermally stable. Based on the thermal analysis results, the temperatures of 400, 500, and 600 °C were selected to perform the heat-treatment process in order to form corn-derived biocarbons, as explained in the following sections.

### 2.2. Structural and Surface Characterizations

**Figure 3:**
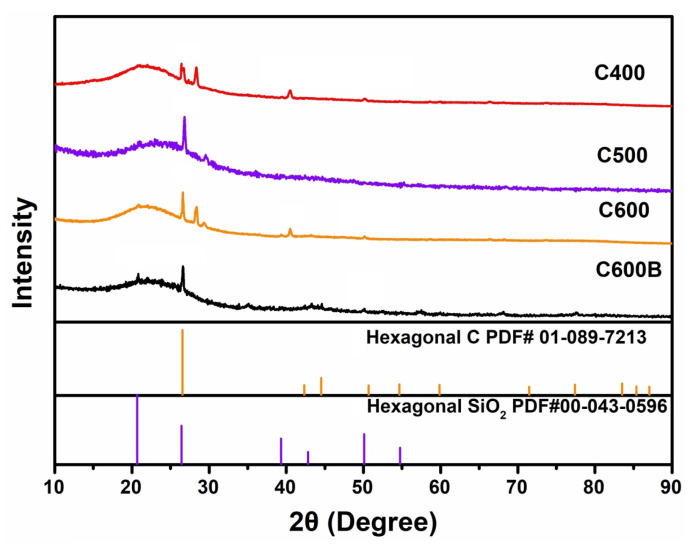
XRD patterns of various samples.

Based on the thermal analysis results, the corn leaves sample was heat-treated at selected temperatures of 400, 500, and 600 °C to prepare biocarbons, called C400, C500, and C600. The latter was ball-milled, and the ball-milled sample was acid-washed to produce C600B. Figure 3 shows the XRD spectra of the samples. It can be observed that there is no obvious diffraction peak in the spectra of all samples, suggesting the semi-amorphous structure of biocarbons. The wide diffraction peaks at about 15–30° correspond to the (002) crystalline domains in the semi-amorphous structure of biocarbons. In addition to the peak corresponding to carbon, other peaks, including the one at ≈26.7°, can be assigned to silica (SiO_2_), as shown in Figure 3. The presence of silica in other agricultural wastes such as rice husk [23] has also been reported. Notably, there are minor peaks related to impurities in samples obtained after the heat-treatment, namely C400, C500, and C600. In contrast, the sample obtained by the mechanochemical treatment of C600, namely C600B, only contains amorphous carbon and SiO_2_, while the peaks related to impurities almost vanish.

**Figure 4:**
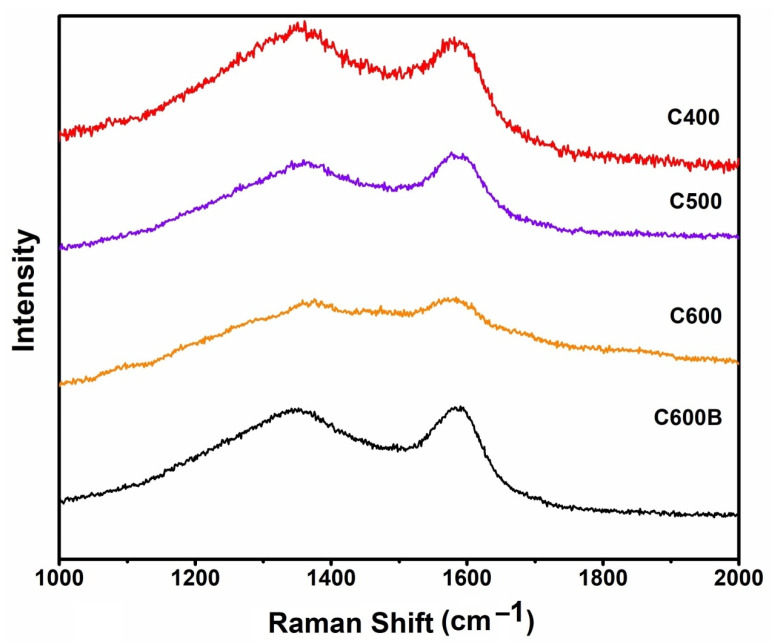
Raman spectra of various samples.

Figure 4 presents the Raman spectra of the biocarbons obtained at various temperatures, and the one obtained after the mechanochemical treatment. The Raman spectra can be characterized by the presence of two characteristic Raman peaks at around 1360 and 1580 cm^−1^, corresponding to the D and G bands, respectively, typically observed in carbon-based materials. The G band is related to the in-plane sp^2^ aromatic domains of the graphitic carbon structures, while the D band indicates the crystalline defects [24,25,26,27] and sp^3^ hybridized carbon [28]. The intensity ratio I_D_/I_G_, which represent the level of crystalline disorder, could be evaluated to be in the range 0.91–0.96, confirming the semi-amorphous nature of the materials.

**Figure 5:**
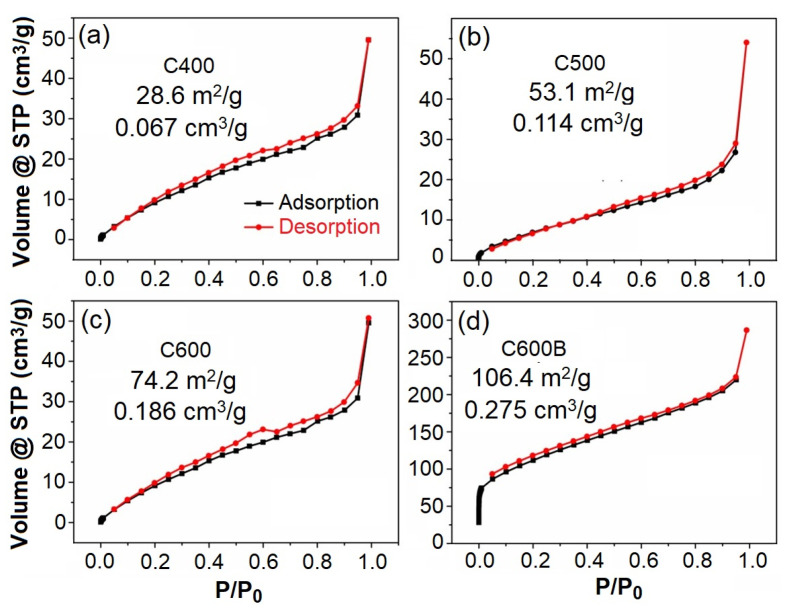
N_2_ adsorption-desorption isotherms of (**a**) C400, (**b**) C500, (**c**) C600, and (**d**) C600B. Values of specific surface area and pore volume are also shown.

Figure 5 exhibits the nitrogen adsorption-desorption isotherms of biocarbons, where all samples show the type IV isotherm with the H_3_ hysteresis loop. These features often represent mesoporous structures [29]. As can be observed, the specific surface area and pore volume of samples increases with the increasing the heat-treatment temperature from 28.643 m^2^/g (pre volume: 0.067 cm^3^/g) obtained at 400 °C to 74.2 m^2^/g (pore volume: 0.186 cm^3^/g) at 600 °C. Moreover, the mechanochemical processing of the C600 sample leads to the further increase of these values to 106.4 m^2^/g (pre size: 0.275 cm^3^/g). The reduced impurity and enhanced surface area and pore volume in C600B leads to the promoted Na-ion storage performance of the material, as discussed later in this article.

### 2.3. Morphological Characteristics

**Figure 6:**
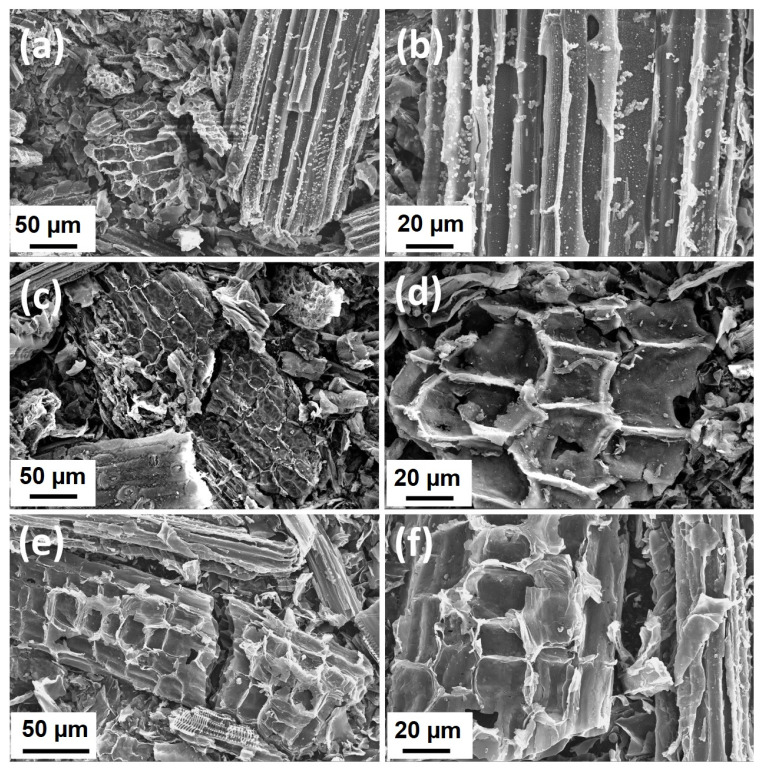
SEM micrographs of (**a**,**b**) C400, (**c**,**d**), C500, and (**e**,**f**) C600.

**Figure 7:**
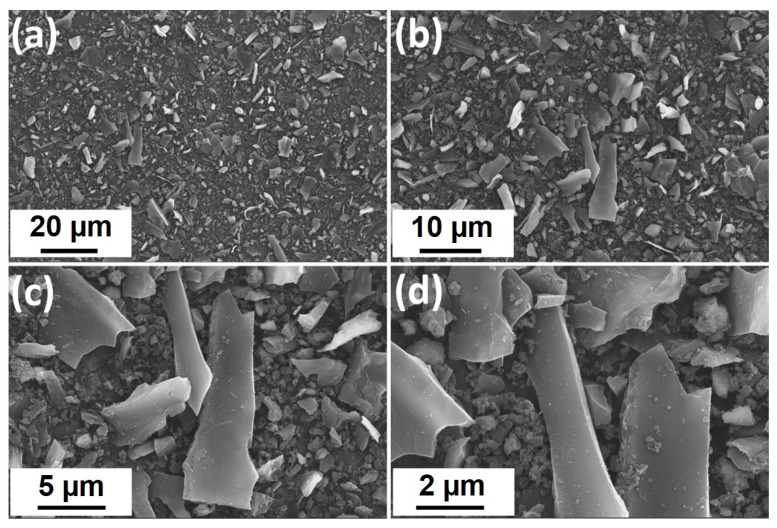
(**a**–**d**) SEM micrographs of C600B at different magnafications.

The biocarbons obtained by the carbonization of the biomass were subjected to SEM study, and the results are shown in Figure 6. The micrographs of C400, C500, and C600 do not show considerable difference, while the morphology is dominated by the presence of strips and honeycomb structures. In comparison with these samples, the morphology of the C600 sample subjected to ball-milling and washing treatment (C600B) is different, in that the particle sizes are substantially reduced to typically less than 20 µm, while the textured particles could no longer be observed (Figure 7). The fragmented particles are indicative of a high-energy ball-milling process [30], which can be beneficial in Na-ion storage application due to the shortening of the Na^+^ diffusion paths and increasing the storage active sites, thus promoting the electrochemical performance of the material [31]. The chemical analyses of C600 and C600B determined by energy dispersive spectroscopy (EDS) are shown in Table 1. As shown, a considerable amount of impurities containing elements K, Mg, Ca, and Si is removed from the biocarbon by the ball-milling acid-washing process. CB600, however, contains a small amount of Al_2_O_3_, originating from the alumina balls used for the ball-milling of C600.

### 2.4. Electrochemical Performance

**Figure 8:**
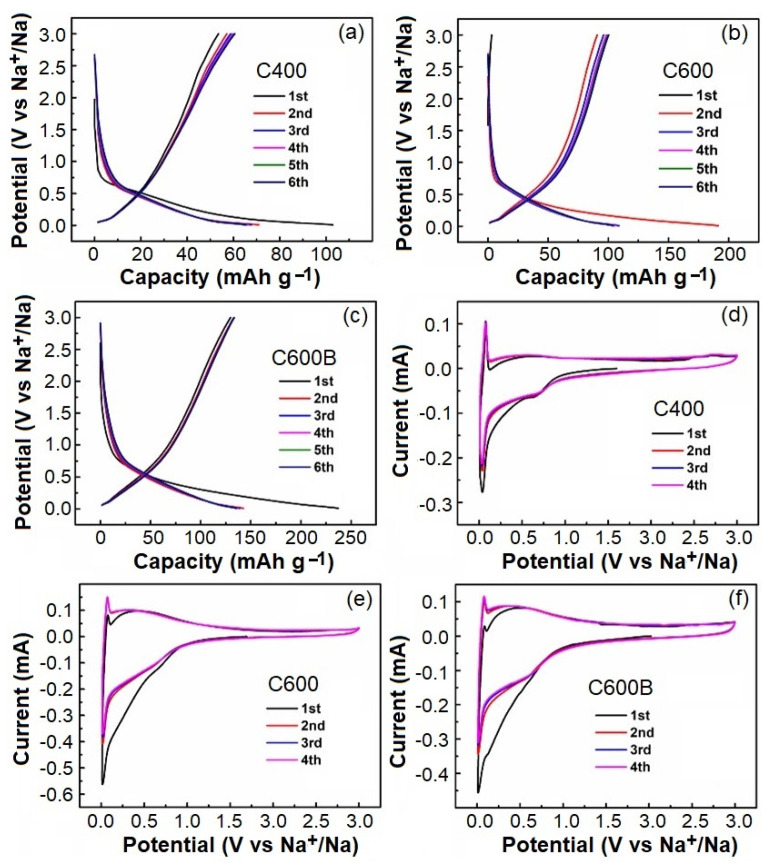
The GCD curves of (**a**) C400, (**b**) C600, and (**c**) C600B. CV curves of (**d**) C400, (**e**) C600, and (**f**) C600B.

Na-ion storage performance of selected biocarbons was studied. Figure 8a–c illustrates the galvanostatic charge discharge (GCD) curves of C400, C600, and C600B at the current density of 500 mA g^−1^. As can be observed, the overall GCD curves of the samples are similar, but the values of reversible capacity gradually increase from C400 to C600 and C600B. For instance, the first cycle-specific charge capacity of C400C is only 53.5 mAh g^−1^, while that of C600 is 93.2 mAh g^−1^, and that of C600B is 129.7 mAh g^−1^. It can be observed that the specific charge capacity of the three materials at the first cycle is considerably lower than the specific discharge capacity, which is due to the formation of solid electrolyte interphase (SEI) film through the decomposition of electrolytes on the electrode and other side reactions, occurred during the first discharge process [32]. However, the charge/discharge curves gradually coincide at larger cycle numbers, indicating the relative stability of SEI films. The results obtained indicate the improvement of the electrochemical performance by increasing the heat-treatment temperature from 400 to 600 °C, and the application of ball-milling/washing process. This improvement can be related to the formation of crystalline nanodomains [33] by increasing the heating temperature, and the reduction of particle sizes, while increasing the purity by applying the ball-milling/washing step.

Figure 8d–f exhibits the cyclic voltammograms (CV) of C400, C600, and C600B conducted within the voltage range 0.01−3.0 V vs. Na^+^/Na at the scanning rate of 0.3 mV s^−1^. In the initial cycle, the CV curves of samples show a reduction peak at about 0.65 V, caused by the formation of SEI film on the electrode. This reduction peak gradually disappears in the subsequent cycles, further confirming the relative stability of the formed SEI layers. The gradual coincide of CV curves in subsequent cycles indicates that the irreversible process and the resulting capacity loss mainly occur in the first discharge/charge process, highlighting the desirable cycle stability and reversibility of the biomass carbons for N-ion storage. Figure 8d–f also exhibits a sharp cathodic event commencing at voltages below 0.5 V, which is intensified at voltages below 0.1 V. This event corresponds to the reversible insertion of Na^+^ into biocarbons. No obvious additional cathodic peak could be observed. The sharp reduction peak mentioned above is accompanied by an anodic event at the low voltage region of 0.01–0.2 V, representing the reversible extraction of Na^+^ out of biocarbons [34].

**Figure 9:**
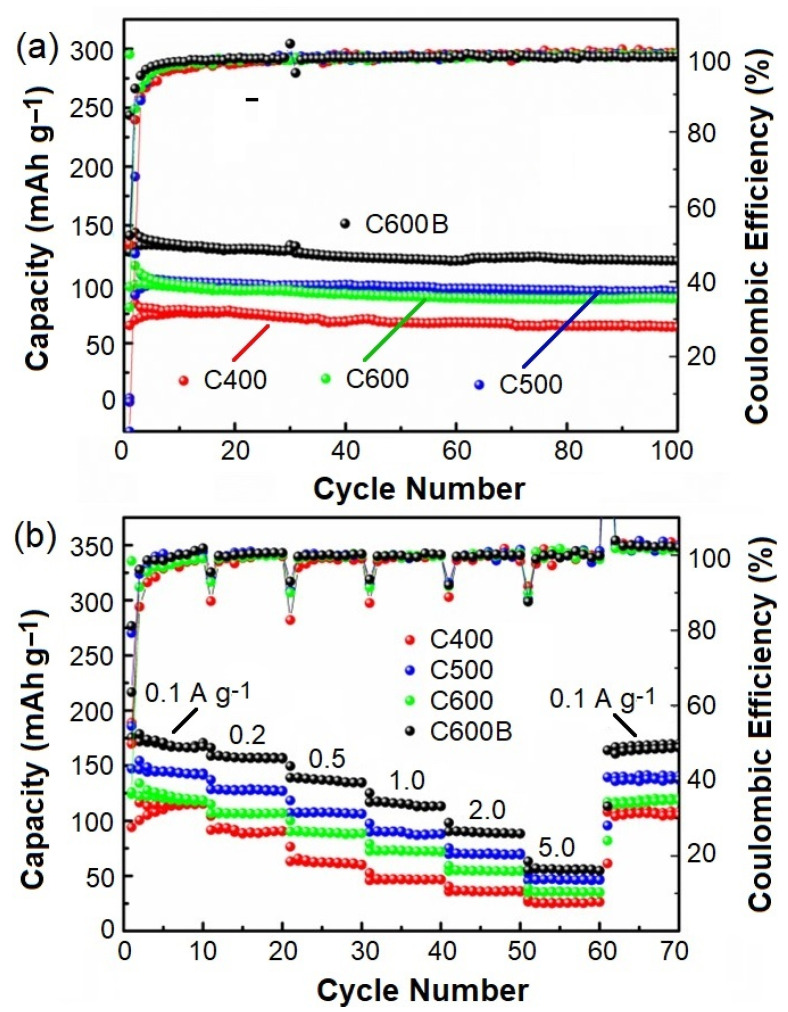
(**a**) Cycling performance and (**b**) rate performance of C400C, C500C, C600C, and C600CB.

The galvanostatic cycling performance of biocarbons was further studied. Figure 9a compares the cycling performances of C400, C500, C600, and C600B recorded at a current density of 500 mA g^−1^. The results obtained indicate the cycling stability of the materials, where the specific discharge capacities of C400, C500, C600, and C600B were recorded to be 78.4, 99.4, 102.3, and 133.7 mAh g^−1^, respectively, for 100 Na-ion insertion and extraction cycles at a relatively high-current density of 500 mA g^−1^. It is evident that the specific capacity of biocarbons is affected by the carbonization temperature, which is in agreement with the thermal analysis results shown in Figure 2. In the DSC curve shown in Figure 2, the exothermic temperature of 445 °C was mentioned to be indicative of the removal of oxygen-containing functional groups, the lignin decomposition, and the reorganization of the carbon structure. As shown in Figure 9, the Na-ion storage performance of C400, obtained by the thermal treatment of the biomass at 400 °C (below the critical temperature of 445 °C), is lower than those of C500 and C600, obtained at 500 and 600 °C, respectively. This improvement can be related to the development of disordered graphitic structure at greater temperatures, providing further active sites to accommodate Na-ions [35]. Moreover, as can be observed in Figure 9a, the specific capacity of C600B is greater than that of other samples, which is related to the higher carbon purity and lower particles sizes in this sample. Figure 9b shows the rate performances of C400, C500, C600, and C600B under various current densities of 100 to 5000 mA g^−1^, and the results obtained are summarized in Table 2, providing evidence for the greater electrochemical performance of C600B.

**Figure 10:**
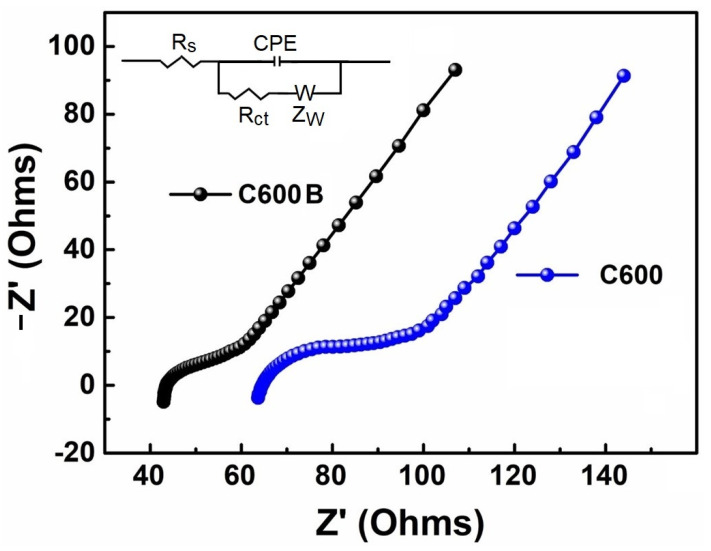
Nyquist plots of the electrode fabricated using (**a**) C600 and (**b**) C600B. Randles circuit is also shown.

Due to enhanced Na-ion storage performances of C600 and C600B, these samples were studied by electrochemical impedance spectroscopy (EIS), and the results are shown in Figure 10. The semi-circles observed in the high frequency region of Nyquist plots represent the charge transfer resistance (R_ct_) at the solid-liquid interface. From Figure 10, the lower R_ct_ value of C600B is evident, which is due to the greater contact points between the electrode and electrolyte and higher carbon purity of the biocarbon. On the other hand, the low-frequency region of Nyquist plots represents the resistance associated with the diffusion of Na-ions into/out of the electrode.

To study the kinetics of charge storage, the Na-ion diffusion coefficient (D_Na_^+^) was calculated from the linear part of the EIS curves, according to Equation (1) [36,37]:

(1) $$D_{{\mathrm{Na}}^{+}}=\frac{R^{2}T^{2}}{2A^{2}n^{4}F^{4}C^{2}\sigma^{2}}$$

In Equation (1), R (8.314 J mol^−1^ K^−1^) is the gas constant, T represents the temperature (K), while n is the molar number of transferred electrons, and A is the apparent surface of the electrode, which is considered to be 1.13 cm^2^. Moreover, F (96,485 C mol^−1^) is the Faraday constant and C is the concentration of Na-ions, which can be obtained by considering the active material’s tapping density [38]. Also, σ is the Warburg impedance coefficient, determined from the slope of the line, derived from the impendence spectra [39]. The equivalent circuit (Randles circuit), which is shown as the inset of Figure 10, includes the electrolyte resistance (R_s_), the charge transfer resistance (R_ct_), the double-layer capacitance (CPE), and the Warburg impedance (Z_w_). Since the rate of the faradaic reaction is often controlled by the diffusion of the reactants to the electrode surface, the Warburg impedance, which indicates the diffusional resistance element, is in series with R_ct_.

Table 3 summarizes the extracted information, including the electrolyte resistance (R_s_) and charge transfer resistance (R_ct_) [40]. As shown, the values of R_s_ and R_ct_ recorded on C600B are lower than those of the C600, while the Na-ion diffusion coefficient of the C600B is greater than that of the C600, which is consistent with the greater cycling performance of the earlier. This highlights the effect brough about by the mechanical milling and washing process.

### 2.5. Pseudocapacitive Performance

**Figure 11:**
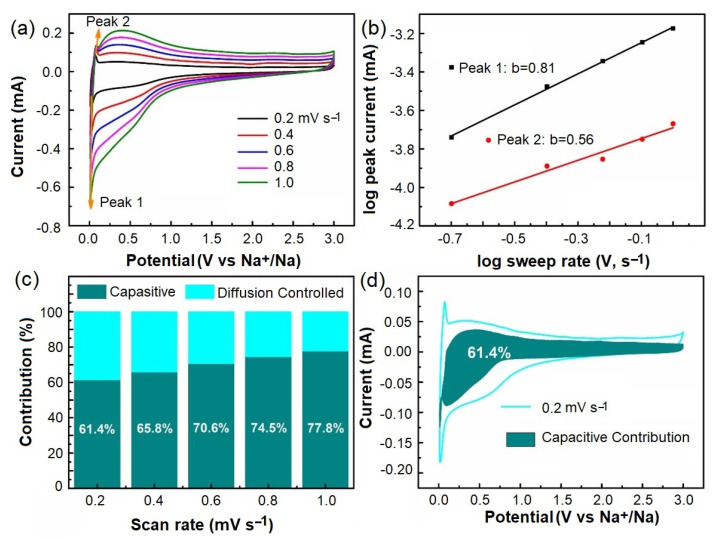
Electrochemical behavior of C600B: (**a**) Cyclic voltammograms recorded at different scan rates. (**b**) Linear plots of log (current) vs. log (scan rate). (**c**) Relative capacitive/diffusion-controlled contribution to the total current at different scan rates. (**d**) Capacitive contribution at the scan rate of 0.2 mv s^−1^.

To analyze the Na-ion storage performance of C600B, the CV curves of the material were evaluated at 0.01–3.0 V vs. Na^+^/Na at scan rates varying from 0.2–1.0 mV s^−1^, as shown in Figure 11a. As observed, the enclosed area in the CV curve increases with the increase of the scanning rate. This observation can be explained by the fact that, at higher scan rates, more ions reach the electrode/electrolyte interface, increasing the capacitive current, whereas limited ions participate in the charge-transfer reaction. In addition, the positions of the reduction/oxidation peaks do not change at various scanning rates, indicating the reversibility [41] of sodium ion insertion/extraction into/out of C600B. The variation of scanning rates and corresponding peak currents can be used to evaluate the pseudocapacitance contribution to the total current at different scan rates. The diffusion-based behavior of the electrode implies that the peak current (i) changes linearly with the square root of the scan rate (v^1/2^). However, if the peak current varies linearly with the scan rate, the process can be assumed to be based on pseudocapacitive behavior. The correlativity between i (current intensity) and v (sweeping rate) can be based on the following equations [42,43]:

i = av^b^ (2)

log i = log (a) + b × log (v)(3)

In Equation (2), i is the peak current. The terms a and b represent dimensionless variables, and v is the scan rate. If b = 0.5, the current response is diffusion-controlled. If the b value is 1, the electrochemical process is based on a pseudocapacitive event. Figure 11b shows the values of log i vs. log v, where the slope of lines corresponding to peak 1 (cathodic event) and peak 2 (anodic event) could be calculated to be 0.80 and 0.56, respectively. This indicates that the cathodic peak is generated mainly based on a pseudocapacitive process, while the anodic event is mainly based on a diffusion process [44]. In addition, Equation (2) can be expressed as follows [45]:

i = k_1_v + k_2_v^1/2^ (4)

(5) $$\frac{i}{v_{1/2}}=k_{1}v^{1/2}+k_{2}$$

where k_1_v and k_2_v^1/2^ correspond to the pseudocapacitive- and diffusion-based contributions of redox ions, respectively [46,47]. From Figure 11c, it can be observed that the pseudocapacitive contribution of Na ions to the total current measured on C600B is 61.4%, 65.8%, 70.6%, 74.5%, and 77.8% at scan rates of 0.2, 0.4, 0.6, 0.8, and 1 mV s^−1^. As can be seen, with the increase of the scan rate, the pseudocapacitive contribution is enhanced, indicating the promoted surface involvement. Even at a scan rate of 0.2 mV s^−1^, the pseudocapacitive contribution to the total current is remarkable at 61.4%, as shown in Figure 11d.

## 3. Discussion

Due to the rising world population, the increasing demand for food is inevitable. Therefore, the intensification of agricultural activities leads to the substantial increase of biomass waste. Among this, corn-based waste is generated in large quantities, greater than 200 million tons per year. The residues of corn production include mainly the corn cob, straw, and leaves, which form around 70 wt% of the total production. For instance, Brazil produces around 82.2 million tons of corn, producing around 60 million tons of waste, which might be used to feed animals or to cover the soil [48,49,50]. Due to its large availability, corn waste can be used as a viable resource for various other applications, such as biochar, used as an adsorbent [51], high-value polyphenols [52], biogas [53], and anode materials for metal-ion batteries.

Cong et al. [54] employed a hydrothermal-assisted route to convert corn stalk powders at 180 °C (12 h), followed by thermal at 1000 °C, to achieve carbonization. The electrode made of the hard carbon material obtained using PVDF binder and NMP solvent exhibited a reversible capacity of more than 200 mAh g^−1^ at the current density of 200 mAh g^−1^. In another work, Zhu et al. [13] employed corn pods as the carbon source to fabricate anodes of Na-ion batteries. The process included heating at 700 °C (2 h) in nitrogen, washing with acids, and treating with chemicals such as Cu(NO_3_)_3_, Co(NO_3_)_2_, NH_4_F, and urea at 120 °C (8 h). The product delivered a specific capacity of 195 mAh g^−1^ after 100 cycles at the limited current density of 200 mA g^−1^.

In contrast with wastes such as corn stalk [54] and corn pods [13], corn leaf has not received attention in the literature as the source of biocarbons. Moreover, further development of simple methods for the conversion of corn biomass into high-value materials is required to provide a sufficient driving force for the commercialization of such materials. Also, the utilization of water-soluble binders, as opposed to PVDF, can enhance the environmental cleansing aspect of the process. It should be mentioned that, though previously considered biologically inert, NMP has been found to demonstrate potential disease-stabilizing and immunomodulatory activity [55,56]. In particular, due to environmental, health, and safety concerns, the European Union has restricted the use of NMP. Therefore, the environmental policies set off the demand for greener alternatives [57].

The current research provides three main new insights: (1) corn leaf can be employed as the carbon source for the preparation of biocarbon, applicable as the anode material for Na-ion batteries; (2) the implementation of simple carbonization in air of corn leaf, followed by a washing step, can lead to the preparation of biocarbons with relatively high specific capacity and high-rate capability; and (3) the successful utilization of water-soluble carboxymethyl cellulose binder was demonstrated.

Thermal analysis was performed, comprising DSC and TGA on corn leaf samples, based on which, two exothermic events were identified at 327 and 445 °C. The first event was related to the thermal decomposition of the hemicellulose of leaves, and the second was related to the decomposition of lignin of the biomass, the elimination of oxygen-containing functional groups, and the restructuring of the carbon phase. Accordingly, the corn leaves were thermally treated in air at selected temperatures of 400, 500, and 600 °C to form biocarbons, and the Na-ion storage performance of the samples was evaluated. It was observed that increasing the temperature from 400 to 500 °C considerably increases the Na-ion storage performances of biocarbons; this was assigned to the formation of graphitic nanodomains, which could provide further active sites to accommodate Na-ions. Increasing the temperature to 600 °C further improves the electrochemical performance of the sample. Furthermore, the application of the ball-milling and washing process would reduce the particle sizes of the carbonaceous material and increase the carbon purity of the sample. This sample provides a Na-ion storage capacity of 134 mAh g^−1^ after 100 cycles at high-current density of 500 mA g^−1^. The pseudocapacitive contribution to the total current measured on the sample was found to be in the range 61–78% at scan rates of 0.2–1.0 mV s^−1^. The results obtained confirmed the application of carbonization treatment at a moderate temperature of 600 °C for a short period of 20 min, followed by a ball-milling process for 2 h and washing, as a simple and potentially green approach to convert corn leaves into biocarbons for low-cost Na-ion storage performance. Future research may provide further economic and environmental evaluation of the proposed method.

It should also be mentioned that carboxymethyl cellulose (CMC) is a linear polymeric derivative of natural cellulose which contains hydroxyl (OH) and carboxymethyl (COOH) groups. Consequently, CMC is a water-soluble binder [58], avoiding the utilization of organic binders such as NMP. Therefore, CMC, with an annual production of ≃2 × 10^5^ tons [59], can be considered as a more environmentally friendly and cost-effective [60] option to be used as a binder of electrodes used in metal-ion batteries. The results presented here show the desirable electrochemical performance of CMC binder in combination with corn-derived carbon. Here, CMC could provide a homogeneous slurry by avoiding the agglomeration of carbon particles and gelation of slurry. Moreover, CMC binder can form a gel polymeric network to enhance the electrical conductivity and integrity of the resultant electrode. According to the literature, CMC has been utilized to improve the electrochemical performances of Si-based compounds [61]. The current work demonstrates the possibility of employing the water-soluble CMC binder to fabricate electrodes made of corn-derived biocarbons. The structural, rheological properties and the gelation behavior of CMC corn-derived carbons in an aqueous environment, and their influence on homogeneity of the slurry and the electrochemical performances of the resultant electrodes, should be further studied in future research.

## 4. Materials and Methods

In this research, corn leaf powder, obtained from a farm located in Shenyang (Liaoning province, China) was used as the biomass. The material was treated with deionized water in order to remove its soluble impurities, followed by vacuum filtering and drying a 90 °C for a few hours. The deride power was employed for various experiments, as explained below.

### 4.1. Thermal Analysis

Thermal analysis, consisting of thermogravimetry analysis (TGA) and differential scanning calorimetry (DSC), were performed on the corn leaf powder using a thermal analyzer (TASDT-Q600) equipped with alumina crucibles, which was used as the sample holder and the reference. The thermal analysis was performed at 20 °C min^−1^ under an air flow rate of 100 mL min^−1^. Based on critical temperatures, obtained temperatures of 400, 500, and 600 °C were selected to contact gram-scale carbonization experiments, as explained below.

### 4.2. Carbonization Trials

The deride sample was placed into a corundum crucible and heated in a furnace to achieve the pyrolysis carbonization of the material. The heat treatment was conducted by raising the temperature from room temperature to various temperatures of 400, 500, and 600 °C at a heating rate of 5 °C min^−1^ followed by a dwell period of 2 h, before the temperature was reduced to room temperature. The samples obtained by heating the corn leaf powder at 400, 500, and 600 °C are called here C400, C500, and C600.

### 4.3. Ball-Milling Acid-Washing

The sample prepared through the carbonization of corn leaf powder at 600 °C (C600, 2 g) was ball-milled using 60 g of alumina balls at a rotational speed of 250 rpm for 2 h using high-energy planetary ball-milling equipment. The ball-milled sample was then magnet-stirred in hydrochloric acid (HCl, 37%) for 30 min at 50 °C in a fume hood, followed by diluting the acid with deionized water, and vacuum filtration. Then, the sample was repeatedly rinsed with water until a pH value of about ≈7 was achieved. The sample was then dried at 100 °C for 12 h. The sample obtained was called C600B.

### 4.4. Structural and Microstructural Characterization

Phase composition of samples was studied using a PANalyco X-ray diffractometer (Malvern Panalytical, Malvern, UK) at 40 KV. The instrument employed Cu-K_α_ radiation (λ = 0.1542 nm) in the 2θ values of 10° to 90°. Raman spectroscopy was employed using a LabRam spectrometer (Horiba, Kyoto, Japan) (HR822.9, 633 nm) at 1000–2000 cm^−1^. Nitrogen adsorption-desorption isotherms were tested using the Barrett-Joyner Halenda (BJH) method, where the specific surface area and the pore size distribution were measured according to the Brunauer-Emmett-Teller (BET) model. The morphological characterizations of samples were evaluated using a Zeiss Ultra Plus field emission scanning electron microscope equipped with an energy dispersive X-ray detector (EDS). The accelerating voltage was 15 kV.

### 4.5. Electrochemical Measurements

The electrochemical performances of corn leaf-derived carbonaceous materials were characterized using various techniques. To evaluate their Na-ion storage performance, the samples were mixed with Super-C45 as the carbon conductive agent and aqueous solution of carboxymethyl cellulose (CMC, 10%) as the binder at the mass ratio of active material: conductive carbon: binder = 7:2:1. The mixture was ground for 30 min, and the uniform slurry obtained was coated on a copper foil to provide a thickness of 150 μm. The electrode sample was then dried at 90 °C for 12 h. The electrodes were assembled into the CR2025 coin half-cells using Na disks as the reference/counter electrode in a glovebox (Mikrouna, Shanghai, China) under high-purity argon. For this, 1.0 M NaPF_6_, in a mixture of EC:DEC:DMC (1:1:1 *w*/*w*/*w*), was used as the electrolyte. The galvanostatic discharge-charge measurements were carried out using a battery testing system (LAND-CT2001A, Landt Instruments, Vestal, NY, USA) at 0.01–3.0 V. Cyclic voltammetry. Measurements were recorded within 0.01 to 3.0 V (vs. Na^+^/Na) at 1.0 mV s^−1^ using a CHI-660E workstation (CH Instruments, Bee Cave, TX, USA). The pseudocapacitive behavior of samples was analyzed by recording CV curves at 0.2–1.0 mV s^−1^. A CHI-660E workstation was used for electrochemical impedance spectroscopy (EIS).

## 5. Conclusions

We have provided evidence that corn leaf can be considered as the carbon source for the preparation of biocarbons applicable as the anode of Na-ion batteries. The thermal analysis of corn leaf samples indicated the presence pf exothermic events at 327 and 445 °C, based on which the corn leaf sample was subjected to carbonization thermal treatment at selected temperatures of 400, 500, and 600 °C. The results showed that the Na-ion storage performances of C500 and C600 outperform that of C400. Moreover, the mechanochemical treatment of C600 (ball-milling and washing) led to the increase of the carbon content of the biomass and the reduction of impurities. At the current density of 100 mA g^−1^, C400, C500, and C600 provided specific capacities of 114, 124, and 144 mAh g^−1^ after 10 cycles. The mechanochemical treatment of C600 improved its electrochemical performance, providing a capacity of 171 mAh g^−1^ after 10 cycles at 100 mA g^−1^. At a promoted current density of 500 mA g^−1^, the treated sample (C600B) provided a Na-ion storage capacity of 134 mAh g^−1^ after 100 cycles. At the high rate of 5000 mA g^−1^, the sample could still provide a capacity of 56 mAh g^−1^ after 60 cycles. The charge-transfer resistance and Na-ion diffusion coefficient of C600B was evaluated to be 49 Ω and 4.8 × 10^−19^ cm^2^ s^−1^. The pseudocapacitive contribution to the total current measured on C600B was found to be in the range 61–78% at scan rates of 0.2–1.0 mV s^−1^. The desirable performance of the electrode could be contributed to a combination of effects, including the presence of fine, semi-amorphous carbon particles well-integrated by gel polymeric networks, obtained through the utilization of a water-soluble CMC binder.

## Figures and Tables

**Table 1:** Chemical composition (wt%) of C600 and C600B.

	C	O	Si	K	Ca	Mg	Al
C600	70.73	19.53	6.67	1.67	1.08	0.33	-
C600B	78.00	18.76	2.58	-	-	-	0.66

**Table 2:** Discharge specific capacity of biocarbons at different current densities.

	Current Density (mA g^−1^)/Cycle Number						
Sample	100/10	200/20	500/30	1000/40	2000/50	5000/60	100/70
C400C	114.2	88.9	62.5	47.0	35.9	25.5	106.7
C500C	124.3	106.9	89.4	73.0	54.9	35.4	116.2
C600C	144.4	127.3	107.7	90.3	69.7	46.3	135.8
C600CB	170.7	157.0	137.0	115.6	89.3	55.6	164.3

**Table 3:** The electrode kinetic parameters obtained from equivalent circuit-fitting of Nyquist plots shown in Figure 10.

	R_s_ (Ω)	R_ct_ (Ω)	σ (Ω s^−1/2^)	D_Na_^+^ (cm^2^ s^−1^)
C600	65.13	85.90	273.31	3.71 × 10^−19^
C600B	42.67	48.89	241.03	4.77 × 10^−19^

## Data Availability

Data will be provided upon request.
